# An open-label, clinical feasibility study of the efficacy of Remdesivir for Long-COVID

**DOI:** 10.1186/s40814-026-01823-9

**Published:** 2026-05-01

**Authors:** Helen Neilens, Victoria Allgar, Kayle-Anne Sands, Jade Chynoweth, Amber Lord, Paigan J. Aspinall, Helen Hambly, Anton Barnett, Lindsay Skipper, Tom Bewick, Ian Maidment, Hairil Abdul Razak, Ruth Ashton, David Strain, Karen Knapp, Mark A. Faghy

**Affiliations:** 1 Peninsula Clinical Trials Unit, University of Plymouth, Plymouth, UK; 2 Medical Statistics Group, University of Plymouth, Plymouth, UK; 3 College of Science and Engineering, University of Derby, Derby, UK; 4 University Hospitals of Derby and Burton NHS Foundation Trust, Derby, UK; 5 College of Health and Life Sciences, Aston University, Birmingham, UK; 6 University of Exeter, Exeter, UK/University of Exeter Medical School, Exeter, UK; 7 Sport and Exercise Sciences, Coventry University, Coventry, UK; 8 School of Sport, Exercise and Health Sciences, Loughborough University, Loughborough, UK

**Keywords:** Covid-19, Remdesivir, Long COVID, Safety, Feasibility, Efficacy

## Abstract

**Background:**

Long COVID (LC) affects around two million people in the UK and over 65 million people globally. Antiviral medications have shown positive effects in reducing the risk of progression to severe disease in high-risk patients during an acute SARS-CoV-2 infection and have improved outcomes in those living with LC. Research testing the feasibility and efficacy of antiviral medications is ongoing, and clinical trials should determine the use of Remdesivir and its impact on LC symptoms, patient-reported outcomes, quality of life and functional status.

**Methods:**

Seventy-two patients ≥ 18 years of age who have a confirmed LC diagnosis will be recruited across two sites to trial Remdesivir_TM_ treatment delivered via intravenous infusion over 5 days. This feasibility study will provide high-quality data to estimate screening rates, recruitment through different pathways, retention and treatment adherence. The study will also determine the definitive trial’s most clinically relevant primary outcome. After a detailed screening process, patient-reported and clinical outcome measures (including EQ-5D-5L, Symptom Burden Questionnaire™ for LC (SBQ™), cardiopulmonary exercise tests (CPET), lung function, biomarkers and inflammatory profiles) will be collected to determine symptoms and impact of their condition, which will be repeated post-treatment. A subset of 20 participants will undergo whole-body parametric Fluorodeoxyglucose (FDG) PET/CT to measure multi-organ metabolic activity. Due to the established physical, cognitive and clinical impairment impacts of LC, patient involvement has been extensively embedded within the design and implementation of all study processes to increase patient safety and delivery.

**Discussion:**

This study will provide data on the feasibility of a trial of 5-day intravenous treatment with Remdesivir for patients with LC. The treatment is already effective in the treatment of patients with acute severe cases of SARS-CoV-2. Remdesivir has an established safety profile and carries no higher risk to patients than standard medical care. The findings will inform the design of a future definitive study.

**Trial registration:**

ISRCTN Number: 72940450.

Date registered: 07/06/2024.

URL: https://www.isrctn.com/ISRCTN72940450.

ClinicalTrials.gov Number: NCT05911906.

**Supplementary Information:**

The online version contains supplementary material available at 10.1186/s40814-026-01823-9.

## Background

Long COVID (LC) is a complex and episodic condition that is associated with over 200 symptoms [1] and can range from a mild persistent illness that impacts some aspects of daily life, to a disabling condition that severely affects functional status and quality of life. There are over 65 million people globally [1] living with LC, which is defined as the continuation or development of new symptoms, three months following COVID-19 infection, with symptoms lasting at least two months, with no other clinical explanation [2]. Prevalence figures of LC are likely underestimated due to widespread withdrawal from, and lack of access to, testing and reporting systems. Surveillance data still highlights a growing burden for public health and one that will continue to impact people’s quality of life and functional status. Currently, there are no established treatments for patients with LC. Whilst some clinician-initiated treatments show promise, they have not been rigorously assessed in controlled clinical trials.

Research and international collaborations have been pivotal in advancing the mechanistic understanding of LC in recent years, but there remains a dearth of knowledge in the total systemic, dynamic and interrelated nature of the pathology of LC that is responsible for a broad heterogeneous symptom presentation. The role of viral persistence and ongoing viral replication has been implicated, and there is growing evidence for its role in driving LC symptomology [3]. SARS-CoV-2 ribonucleic acid (RNA) and protein have also been observed in a range of tissue types collected weeks or months after acute coronavirus disease 2019 (COVID-19) [4]. This includes brain regions, lymph nodes, the thorax, the sciatic nerve, ocular tissue, the cervical spinal cord, the brainstem, and the olfactory nerve [5]. The brain and the brainstem are particularly vulnerable to acute and chronic damage from various sources, particularly viral invasion, inflammation, and vascular activation [6]. Data from single-molecular arrays for high-sensitivity detection of low-abundance proteins can detect SARS-CoV-2 antigens in serum, and one-year post-infection, around 60% of patients with Long COVID demonstrated the presence of SARS-CoV-2 spike proteins and a greater number of organ systems involved in symptoms, which were not evident in controls [4, 6, 7].

The role and importance of viral persistence in the pathology of LC serve as justification for the use of antivirals in the treatment of LC, and ongoing research demonstrates potential efficacy. Antiviral medications such as Remdesivir have been demonstrated to be effective in reducing the risk of progression to severe disease in high-risk patients during an acute SARS-CoV-2 infection [8], and in immunocompromised hospitalised patients with persistent viraemia. Remdesivir is licensed for use during an acute admission to hospital for patients with COVID-19 and has demonstrated positive patient outcomes and reduced risk during acute illness and improved long-term outcomes [9]; however, it has yet to be tested in patients who have LC who were not hospitalised during the acute stages of infection. Remdesivir was chosen as the Investigational Medicinal Product (IMP) for this trial due to the positive outcomes during the acute phase of infection (i.e. reduced likelihood of prolonged/persistent symptoms). Work is ongoing in the United States of America to determine the efficacy of alternative antiviral medications, including Paxlovid (NCT05595369), nirmatrelvir [10] (NCT05668091) and ritonavir (NCT05668091).

Individuals with LC are a clinically vulnerable group and have many symptoms that can be exacerbated by any physical and/or cognitive effort. Personal safety must be considered so patients are assured that they are not at risk by participating in the trial. With the support of patient and public representatives, risks have been identified and addressed in the development of this protocol.

Accordingly, there is a need to assess the feasibility of using an antiviral in LC before commencing a double-blind, multi-centre randomised controlled trial to determine its effectiveness. The resulting study will inform treatment decisions that can potentially reduce Long COVID and its severity, improve patient outcomes and restore quality of life.

## Method

### Objectives

The aim of this study is to inform the design and delivery of a future definitive trial to compare the effectiveness of a 5-day treatment of Remdesivir with treatment as usual (TAU) for patients with LC. This feasibility study has the following objectives (please see Table 1: objectives and outcome measures):

**Table 1:** Tabulated summary of objectives and outcomes

	**Outcome measures**	**Timepoint(s) of evaluation of this outcome measure (if applicable)**
**Feasibility objectives**		
Rates of screening	Number of patients screened overall and by centre	Initial screening and detailed screening
Rates of recruitment	Number of patients consented (as a proportion of patients screened, overall and by centre)	Measured at initial screening, detailed screening and baseline
Retention in the study	1. Number of recruited patients completing outcome measures 2. Number of patients attending each appointment 3. Number of participants completing the intervention 4. Number of participants who withdraw at different stages of the trial	1 and 2. Baseline, CPET visits and post-intervention visit (days 0, 7 and 8, 44, 51 and 52 (11 and 55 at PET/CT site)) 3. Number of patients completing each day of treatment 4. Baseline, CPET visit, treatment visit and post-intervention visits (days 0, 7 and 8, 14–22, 44, 51 and 52 (11 and 55 at PET/CT site))
Adherence to the treatment regime of Remdesivir	Attend clinic appointments and complete each treatment session	Days 14–22 during the treatment phase
Completeness of study assessments	Attend appointments and complete assessments	CPET, bloods (and PET/CT at one site)
Completeness of data collection activities	1. Participants complete all patient-reported measures 2. Number of participants who complete physical and physiological tests	Baseline, CPET visit and post-intervention visits (days 0, 7 and 8, 44, 51 and 52)
**Secondary objectives**		
** Pre- and post-intervention patient-reported outcome measures**		Day 0 and + 28 days after IMP (day 44) ^*^Days 7, 8, 51, 52 (Outcome measures on CPET days also)
Quality of life	EQ-5D-5L	
Functional status	PCFS Impact on daily life subscale of SBQ™-LC	
Physical symptoms	MRC Dyspnoea Scale	
Cognitive symptoms	PDQ-5	
Emotional symptoms	GAD-7	
Symptom burden	DSQ-PEM	
	SBQ™-LC^*^	
	FAS^*^	
	MFIS^*^	
**Clinical assessments**		Day 0 and + 28 days after IMP (day 44) ^*^Days 7, 8, 51, 52 (Outcome measures on CPET days also)
Physiological function and physical function	Maximum inspiratory and expiratory mouth pressure (MIP and MEP) Lung function Blood pressure^*^ Oxygen saturation^*^ Breathing rate^*^ Resting heart rate Body temperature^*^ 6MWT, Borg 6–20 and SPO_2_	
Biomarker and inflammatory profiles	FBC CRP D-dimers IL-6 IL-16 IL-18 PCT IFN-γ TNF-α VEGF-D HLA-DP Vitamin D	Days 0, 7, 8, 44, 51, 52
Tolerance to physical stimulus (CPET)	VT1 RCP V̇O2peak End-tidal CO_2_	Days 7, 8, 51, and 52
Daily symptoms and heart rate variability (visible)	Symptom Score Inventory Heart rate variability	Day 0 to day 52
Clinical safety and tolerance parameters of the use of Remdesivir	AE/SAE/AR/SAR/SUSAR	Consent to day 52 (55 if having PET/CT scan)
(one site only) Microvascular function: whole-body FDG uptake using PET-CT methods	Standardised uptake volume (SUV) and Ki of [11] FDG uptake observed during PET/CT scans	Days 11 and 55

*The outcome measures on CPET days only

#### Trial feasibility objectives

- To ascertain screening and recruitment rates (overall and by different recruitment pathways).
- Retention and dropout rates (due to the treatment and/or trial demands, overall and by centre).
- Adherence to treatment regimen (attendance at the hospital clinic to receive 5 days of IMP).
- Completeness of study assessments (cardiopulmonary exercise test (CPET), bloods and positron emission tomography (PET) and computed tomography (CT) [PET-CT]).
- Completeness of all data collection activities, including baseline and + 28 days after treatment.
- Acceptability of outcome measurements (measured by completion rates).

#### Secondary objectives

- To identify the most clinically relevant primary outcome for the definitive study including quality of life; functional status and symptom burden; tolerance to physical stimulus; exercise tolerance and reduced post-exertional symptom exacerbation following incremental exercise; physiological function; physical function; cognitive function and emotional status and/or capacity; biomarker and inflammatory profiles and metabolic activity (whole-body FDG uptake (FDG) uptake using PET-CT methods).
- To determine the clinical safety and tolerance parameters of the use of Remdesivir in the treatment of patients with LC.

### Patient-reported outcome measures

- *Quality of life:*

*Health related quality of life (EQ-5D-5L) *is routinely used in the assessment of the quality of life in respiratory research and is available in more than 130 languages. It comprises five dimensions (previously 3): mobility, self-care, usual activities, pain/discomfort, and anxiety/depression. Each dimension has five levels: no problems, slight problems, moderate problems, severe problems, and extreme problems.

- *Functional status:*

*Post COVID Functional Status Scale (PCFS, impact on daily life subscale of the LC Symptom Burden) *will be assessed to monitor direct recovery and to assess functional sequelae. The PCFS will evaluate the ultimate consequences of COVID-19 on functional status and supplement other instruments that measure the quality of life, tiredness, or dyspnoea in the acute phase. The PCFS covers the full spectrum of functional outcomes and focuses on both limitations in usual duties/activities and changes in lifestyle in six scale grades. Briefly, grade 0 reflects the absence of any functional limitation, and the death of a patient is recorded in grade D. Upward of grade 1, symptoms, pain or anxiety are present to an increasing degree. This has no effect on activities for patients in grade 1, whereas a lower intensity of the activities is required for those in grade 2. Grade 3 accounts for the inability to perform certain activities forcing patients to structurally modify these. Finally, grade 4 is reserved for those patients with severe functional limitations requiring assistance with activities of daily living (ADL).

- *Symptom burden:*

*Symptom Burden Questionnaire for Long COVID (LC symptom burden) *(SBQ™-LC) is a patient-reported outcome (PRO) measure and multi-domain item bank that has been developed to measure symptom burden in adults with “post-acute sequelae of COVID-19" (PASC). The SBQ™-LC is composed of 17 independently functioning, unidimensional scales. Sixteen scales measure symptom burden (i.e. symptom presence, severity, or frequency) across different symptom “domains” and one scale measures symptom impact on daily life.

*Fatigue Assessment Scale (FAS) *is a 10-item scale evaluating symptoms of chronic fatigue. In contrast to other similar measures (e.g. the Multidimensional Fatigue Inventory), the FAS treats fatigue as a unidimensional construct and does not separate its measurement into different factors.

*Modified Fatigue Impact Scale (MFIS)* is a 20-item patient-reported scale designed to measure fatigue across five scales (general fatigue, physical fatigue, reduced activity, reduced motivation, and mental fatigue) [12]. The MFI-20 has been used to assess fatigue in patients with different respiratory conditions, including COPD, pulmonary fibrosis, and lung cancer.

- *Physical function:*

*Medical Research Council (MRC) Dyspnoea Scale *is scale to grade how breathless a patient gets doing everyday activities; the scale contains 5 response options, ranging from “I only get breathless with strenuous exercise” to “I am too breathless to leave the house”.

- *Cognitive function:*

*Perceived Deficit Questionnaire (PDQ-5)*: The full-length PDQ consists of 20 items and provides a self-report measure of cognitive dysfunction. This instrument provides an assessment of several domains of cognitive functioning that are frequently affected in MS: attention, retrospective memory, prospective memory, and planning and organisation.

- *Psychological:*

*Generalised Anxiety Disorder (GAD-7) *a seven-item diagnostic tool validated in both the primary care setting and the general population. The GAD-7 is a 7-item scale that reports scores from 0 to 3 on all the questions. It investigates how often the patient has been bothered by seven different symptoms of anxiety during the last two weeks with response options such as: “not at all”, “several days”, “more than half the days”, and “nearly daily” scored as 0, 1, 2, and 3, respectively. The scores of 5, 10, and 15 are taken as cut-off points for mild, moderate, and severe anxiety, respectively.

- *Symptom frequency:*

*Modified De Paul Symptom Questionnaire-Post Exertional Malaise (DSQ-PEM)* assesses symptom frequency and severity over a 6-month look back period, however, for the purposes of this study, it will be modified to assess over a 1-week look back period (in-line with Zimmerman et al.: Clinical Trials Identifier: 05595369). Frequency is rated on a 5-point Likert scale: 0 = none of the time, 1 = a little of the time, 2 = about half the time, 3 = most of the time, and 4 = all the time. Severity is also rated on a 5-point Likert scale: 0 = symptom not present, 1 = mild, 2 = moderate, 3 = severe, and 4 = very severe. Post-exertional malaise (PEM) will be deemed to be present if the participant reports having at least one moderate (rated severity ≥ 2) PEM symptom for a frequency rated ≥ 2.

### Design

This is a phase IV, open-label, single-arm, proof-of-concept study.

#### Trial setting

The trial is using two university sites for recruitment and testing participants, and two National Health Service (NHS) trusts as trial treatment sites. One NHS Patient Identification Centre (PIC) is involved. For more information, contact the author.

### Eligibility criteria

#### Inclusion criteria

Patients must satisfy all the following criteria to be enrolled on the study:

-  ≥ 18 years of age at the time of enrolment.
- Previously confirmed or suspected SARS-CoV-2 infection.
- Confirmed diagnosis* of LC by a Health Care Practitioner according to the definition provided by the World Health Organisation [2] for persistent symptoms following a confirmed SARS-CoV-2 infection.
- Evidence of a persistent symptom profile relative to pre-COVID-19 status as derived from patient-reported outcome measures.
- Willing and able to provide informed consent, complete the surveys, complete all planned clinical assessments, and return for scheduled study visits.
- Lives within a commutable distance from the relevant site, at the discretion of the local principal investigator (PI).

*World Health Organisation (WHO) define LC as the continuation or development of new symptoms 3 months after the initial SARS-CoV-2 infection, with these symptoms lasting for at least 2 months with no other explanation.

#### Exclusion criteria

Eligibility criteria have been established in conjunction with the guidance set out by the National Institute for Health and Care Excellence (NICE), British National Formulary (BNF) and the summary of product characteristic documentation.

Patients who meet any of the following criteria will be excluded from study participation:

- Treatment history of Remdesivir, molnupiravir, paxlovid and/or any other COVID-19 antiviral medication (< 6 months).
- Confirmed compromised immune system/function.
- Currently engaged in a physical rehabilitation programme or intervention aimed at improving LC symptom profile and/or functional status.
- Recognised as a ‘severe risk’ of experiencing post-exertional malaise following engagement in physical tasks. Determined using the De Paul Symptom Questionnaire (total score).
- Lack of mental capacity to provide informed consent.
- Unable to understand verbal English/have a hearing impairment that prevents adequate communication*.
- Participation in another clinical drug trial within the last 6 months.
- Currently pregnant, breastfeeding or attempting to get pregnant (i.e. not using effective methods of contraception).
- History of hepatic or renal impairment (estimated glomerular filtration (eGFR; < 30 mL/min) and liver function tests (LFTs) alanine aminotransferase (ALT) > × 5 upper normal limit (ULN)).
- Currently taking medications known to have an interaction with Remdesivir (e.g. chloroquine phosphate or hydroxychloroquine) as defined by BNF information on the selection, prescribing, dispensing and administration of medicines: https://bnf.nice.org.uk/interactions/remdesivir/
- History of serious adverse reactions to medications/infusions used in the study.
- Scan participants only: No recent/long-standing history of CT (within 3 months)/ongoing radiotherapy treatment. Risks of accumulative burden are to be discussed as part of study involvement but are at the discretion of participants.

* English comprehension: Potential participants who are unable to understand verbal English are not eligible for this study. This is due to the necessity of telephone contact which is a key aspect of this study and the unavailability of validated questionnaires in languages other than English.

*Hearing impairment:* Unfortunately, if the participant has a hearing impairment that prevents adequate communication on the telephone, they will not be able to take part in the study. This is clearly stated in the participant information sheet.

### Recruitment and consent

The Trial Management Group (TMG) will closely monitor recruitment performance at each site. The stages of the recruitment process are illustrated in Additional file 1.

#### Participant identification

There are multiple routes being tested in which potential participants may be identified and recruited onto the study to facilitate target recruitment rates, identification and recruitment from clinics and self-referral.

1. Long COVID Centres clinics

Clinical teams at LC clinics will screen existing and new referrals to the service to determine eligibility. Lead Local Collaborators (LCCs) will be responsible for promoting the study amongst relevant staff at the centres to optimise participant identification.

If potential participants are interested, they will be provided with a Participant Information Sheet (PIS) describing the study and including contact details of the local ERASE-LC research team, who they can contact if they have any questions or alternatively their details can be passed on if they prefer.

2. Self-referral

i. Database

Following previous involvement in LC research undertaken on social media, patients have given consent to be contacted about involvement in future research trials. This database contains approximately 1000 potential participants who have contacted the research team directly via email and/or telephone to enquire about interest in participating in the study.

Participants on the database who live within a commutable distance from one of the sites will be contacted about participation and sent a PIS. If they are interested in taking part, they will be asked to complete an Expression of Interest (EOI) form on the website.

ii. ERASE-LC website, social media, posters and leaflets

Social media will be used to promote the study and guide patients with LC to the ERASE-LC Website. Leaflets and posters will also be placed in the clinical areas where patients with LC attend their appointments to direct patients to the ERASE-LC website. Quick Response (QR) codes will be used on documents so potential participants can access the information digitally immediately. The ERASE-LC website will provide information about the study, and the PIS. Patients that are interested may complete the EOI form or contact the local research team directly using the contact details provided on the localised PIS.

The poster and leaflets will also be available in a digital format, and sites will be encouraged to disseminate them via their communication routes, such as newsletters, and share them on their social media accounts.

#### Screening and eligibility

Details of screening and visits are illustrated in Table 2: Standard Protocol Items: Recommendations for Interventional Trials (SPIRIT) figure—tabulated summary of the study data collection by time point.

**Table 2:** SPIRIT figure: tabulated summary of the study data collection by time point

	Screening		Pre-intervention			Treatment phase	Post-intervention			
	Initial screening	Detailed screening	Enrolment and baseline assessment	CPET	PET/CT scan^*^		Safety blood check	Post-intervention assessment	CPET	PET/CT scan^*^
Day			Day 0	Days 7 and 8	Day 11	Days 14–22#	Day 27	Day 44 (+ 28 days after IMP)	Days 51 and 52	Day 55
Informed consent		X	X							
Demographics		X								
Medical history		X	X							
Compliance			X	X	X	X	X	X	X	X
AE assessments			X	X	X	X	X	X	X	X
IMP administration						X				
Pregnancy testing +		X	X	X	X	X	X	X	X	X
PROMS			X					X		
Symptom burden				X					X	
DSQ-PEM	X		X					X		
Physiological and functional status			X					X		
Exercise markers				X					X	
Safety bloods		X					X			
Blood markers			X	X				X	X	
Symptom tracking			X	X	X	X	X	X	X	

^*^Denotes sub-group of participants that will undergo PET-CT scans at the University of Exeter. # denotes range in days to allow for consecutive administration on weekdays. + denotes pregnancy tests that will be administered to WOCP. PROMS: EQ-5D-5L, PCFS, SBQ™-LC, MRC Dyspnoea, PDQ-5, GAD-7, FAS, MFIS. Symptom burden: SBQ™-LC, FAS, MFIS. Physiological and functional status: MIP, MEP, lung function, blood pressure, SpO_2_ measures, heart rate, breathing rate, temperature, Borg RPE, 6MWT. Exercise markers: V̇O2peak, VCO2, RER, blood lactate, blood gas tension, VT1, VT2, RCP, VE, VE/VCO2, ET-CO2. Safety bloods: eGFR, LFTs Blood markers: FBC, CRP, D-dimers, IL-6, IL-16, IL-18, PCT, IFN-γ, TNF-α, VEGF-D, HLA-DP, vitamin D

1. Initial screening

The research team undertakes initial screening by telephone. Data relating to eligibility and screening is collated within the trial master file (TMF) to ensure reporting is in line with the consolidated standards of reporting trials (CONSORT) guidelines.

The researcher captures the pseudonymised details of screened patients in REDCap (Research Electronic Data Capture).

If the participant meets the eligibility criteria, as far as can be determined during the phone call, the researcher will then schedule a face-to-face visit for a detailed screening assessment.

2. Detailed screening visit

The participant attends an appointment at the site located nearest to them at the allocated time to be seen by the research team. A total of 60 min should be allowed for the face-to-face screening visit. This will consist of confirming initial eligibility (20 min), receiving informed consent (10 min) and clinical testing (30 min).

i. Eligibility confirmation

This is conducted by a suitably trained member of the research team. Inclusion criteria from the initial screening will be confirmed. A combination of discussion with the participant and use of medical notes may be used to confirm eligibility. If the medical notes cannot be accessed (e.g. the patient is not registered at the linked Trust), the patient must provide evidence of an LC diagnosis from a healthcare professional to proceed. Medically trained staff need to countersign all eligibility criteria. This is monitored centrally by Peninsula Clinical Trials Unit (PenCTU).

Further eligibility check:

- Evidence of a persistent symptom profile relative to pre-COVID-19 status as derived from patient-reported outcome measures.
- Safety bloods—history of hepatic or renal impairment (eGFR < 30 mL/min) and LFTs ALT > × 5 ULN).
- Pregnancy test.
- PET/CT scan participants only: Recent/long-standing history of CT (within 3 months)/ongoing radiotherapy treatment. Risks of accumulative burden are to be discussed as part of the study involvement but it is at the discretion of the participants.

ii. Informed consent

Patients will have had time to consider and discuss the study with their clinicians, family or friends. If they agree to take part, written formal consent will be taken from the potential participant by the PI or a delegated individual.

The informed consent form includes optional consent for storage of blood samples for use in future ethically approved research and to be contacted about future research opportunities. Where applicable, participants are also given the option to opt out of the PET-CT scans.

iii. Demographics and medical history

Once consent is provided, the following data will be taken as part of the detailed screening:

- Demographics
- Medical history
- LC symptoms (those consistent with LC will be collected to include the most common listed below. This list is not exhaustive as there are over 250 documented symptoms recorded).

Symptoms include extreme tiredness (fatigue), feeling short of breath, muscle aches, problems with memory and concentration ("brain fog"), chest pain or tightness, difficulty sleeping (insomnia), heart palpitations, dizziness, pins and needles, joint pain, depression and anxiety, tinnitus, earaches, feeling sick, diarrhoea, stomach aches, loss of appetite, high temperature, cough, headaches, sore throat, changes to sense of smell or taste, rashes, any others. 

Concomitant medications:

All medications, including over-the-counter or prescription medications, vitamins and/or herbal supplements.

Patients taking medications that are known to interact with the IMP will be excluded from the study. These are documented within the summary of product characteristics (SmPC) and established libraries (BNF/European Medicines Agency (EMA)) which will be reviewed every quarter during screening and recruitment to ensure current safety practices for patients (this includes the following medications: Apalutamide, Carbamazepine, Chloroquine, Enzalutamide, Fosphenytoin, Hydroxychloroquine, Mitotane, Phenobarbital, Phenytoin, Primidone, Rifampicin).

Results from blood tests will be available in approximately 5 days. Patients who are still eligible will be notified and will receive a phone call or email (as arranged) from the research team to confirm the baseline assessment visit.

### Baseline visit

Participants are required to attend the baseline visit within 1 month of their detailed screening. Participants will be sent their Patient Reported Outcome Measures (PROMs) by email 24 h before their baseline appointment to allow more time for them to complete them: EuroQol Group 5 Dimensions 5 Level (EQ-5D-5L) Post-COVID-19 Functional Status (PCFS) [13], Symptom Burden Questionnaire™ for Long Covid (SBQ™-LC) [14], Medical Research Council (MRC) Dyspnoea Scale [15], 5-item Perceived Deficits Questionnaire (PDQ-5) [16], Generalised Anxiety Disorder 7-item (GAD-7) [17], DSQ-PEM, Fatigue Assessment Scale (FAS) [18], and Modified Fatigue Impact Scale (MFIS) [11]. For those requiring paper questionnaire booklets, these will be handed to the participant at the preceding visit with instructions not to complete the questionnaires until 24 h before the baseline visit. A text will be sent to all participants to remind them about their baseline visit and to complete the required questionnaires 24-h beforehand.

In addition to returning the PROMs (if completed on paper), they are required to complete clinical tests for physical function, lung function, physiological function, and biomarker and inflammatory profiles. Participants will also be instructed on how to track their symptoms using an app (Visible) on their phone.

#### Physical function

*Maximum respiratory mouth pressures:* Inspiratory and expiratory mouth pressures and lung function testing will be conducted and analysed in accordance with published and standardised test criteria from the European Respiratory Society (ERS)/American Thoracic Society. Maximum inspiratory (MIP) and expiratory (MEP) mouth pressure (cmH^2^O) will be assessed using a hand-held mouth pressure meter (RP Check, Medical Diagnostics, Kent, UK). Measurements will be initiated from residual volume (MIP) and total lung capacity (MEP), to determine an index of global inspiratory and expiratory muscle strength as described previously [19].

*Lung function testing:* Lung function testing will be assessed using a pneumotachograph (MS03, Micro Medical, Buckinghamshire, UK) to determine forced expiratory volume in one second (FEV1), forced vital capacity (FVC), peak expiratory flow (PEF), FVC/FEV1 and will be conducted in line with the ERS and American Thoracic Society Guidelines [20].

#### Physiological function

*Physiological observations* conducted included blood pressure (millimetres of mercury (mmHg)), oxygen saturation (%), breathing rate (breaths/minute), resting heart rate (beats per minute (bpm)) and body temperature (°C)*.

*Six-minute walk test (6MWT)*: A standardised and widely used measure of functional status in individuals with diseases such as chronic obstructive pulmonary disease, cystic fibrosis, congestive cardiac failure, peripheral vascular disease, and the elderly will be used. It has been previously used to assess responses to interventions and predict morbidity and mortality. Following completion of the 6WMT, patients will provide their rating of perceived exertion (Borg rate of perceived exertion (RPE) 6–20 scale), and their saturation of peripheral oxygen (SPO_2)_ will be recorded. The number of times (frequency, number) and duration (seconds) a patient stops, and the number of metres completed in the allotted time will be recorded.

#### Biomarker and inflammatory profiles

The following biomarkers have been selected because they have previously been demonstrated to correlate with a persistent reduction in quality-of-life scores. They include:

Full blood count (FBC) to determine neutrophil-to-leukocyte ratio (NLR) and the polymorph lymphocyte ratio (PLR), C-reactive protein (CRP), D-dimers, interleukin-6 (IL-6), IL-16, IL-18, procalcitonin (PCT), interferon-gamma (IFN-γ), tumour necrosis factor-alpha (TNF-α), vascular endothelial growth factor D (VEGF-D), human leukocyte antigen-DP (HLA-DP)*, and vitamin D.

*A transplantation antigen of major histocompatibility complex (MHC) class II category of the MHC of humans. It is a heterodimeric cell-surface glycoprotein comprised of an α (heavy) chain and a β (light) chain.

#### Continuous symptom profiling and tracking

All patients will undertake continuous symptom monitoring. Those who have access to a smartphone will do this using an iPhone Operating System (IOS) and Android-compatible application that has been specifically developed for Long COVID. Visible is General Data Protection Regulation (GDPR) compliant, with data being stored within a cloud-based server in Europe. Patients will be asked to create an account and, as part of the Visible App, they will have to complete a personalised 18-item Symptom Score Inventory which tracks the daily impact of different physical and cognitive LC symptoms. Participants will be provided with instructions.

The application also has features that allow continuous monitoring of heart rate variability. To enable this feature, patients will be provided with a CE-marked wearable sensor (Polar Verity Sensor, Polar, Kempele, Finland) that is worn on the upper arm and uses optical technology to record heart rate and heart rate variability.

Participants who do not have access to a smartphone will complete a daily paper symptom diary. The diary should be returned on the day 52 visit, and the data will be uploaded to the electronic case report form (eCRF) by the local research team. Heart rate variability data will not be collected for these participants.

### Cardiopulmonary exercise test (days 7, 8, 51 and 52)

Seven days after their baseline visit, participants will attend a CPET on two consecutive days, which will be repeated post-intervention. They will receive an email and a text reminder 24 h before each CPET day. This will also include the PROMs: SBQ™-LC, FAS and MFIS to complete in advance to provide participants extra time to complete these. A text reminder of the appointments will also be sent.

On arrival at each session, pregnancy tests will be conducted with a urine dip.

Bloods will be taken and biomarkers and inflammatory profiles will be measured. Once patients are ready, they will progress with the CPET.

The 2-day CPET protocol has been specifically designed to determine impaired cardio-respiratory and muscular physiology in patients who are at risk of post-exertional malaise/post-exertional symptom exacerbation [21].The test uses a non-maximal design which allows for the detection of key ventilatory, cardiovascular and perfusion parameters to be observed whilst also imposing a strict inclusion/test termination criterion. The protocol also consists of different strata, and the chosen protocol is determined relative to a patient’s current ability to undertake functional tasks:

- Strata 1: Starting intensity of 10 watts with a 5-watt-per-min increase for 12 min (max intensity 70 watts). This protocol is used for participants who achieve < 350 m on the 6MWT.
- Strata 2: Starting intensity of 20 watts with a 5-watt-per-min increase for 12 min (max intensity 80 watts). This protocol is used for participants who achieve 350–400 m on the 6MWT.
- Strata 3: Starting intensity of 30 watts with a 5-watt-per-min increase for 12 min (max intensity 90 watts). This protocol is used for participants who achieve > 400 m on the 6MWT.

CPET will be performed/overseen by experienced and accredited clinical exercise physiologists. Tests will be used to determine peak oxygen uptake (V̇O2peak) and parameters representative of cardiovascular reserve [22]. CPETs will include continuous/regular monitoring of standard measurements, including inspired/expired air, ventilation profile, 12-lead electrocardiogram (ECG), blood pressure, blood lactate and blood gas tensions (where possible). The latter two measures have relevance to LC populations due to the established hypothesis of impaired mitochondrial function and abnormal aerobic contribution to exercise [23]. Blood samples will be obtained via capillary sampling (finger-prick). These measures are standard protocol as outlined by the British Association of Sport and Exercise Sciences (BASES) [24] and the American College of Sports Medicine (ACSM) [25]. Tests will be conducted using a standard bicycle ramp protocol following American Thoracic Society guidelines [26].

Key variables of interest include:

- The first ventilatory threshold (VT1).
- The respiratory compensation point (RCP).
- V̇O2peak.
- End-tidal CO_2_.

Exercise termination criteria:

- Chest pain suggestive of ischaemia.
- Fall in systolic blood pressure > 20 mmHg from the highest value during exercise.
-  > 225 mmHg systolic blood pressure.
-  > 130 mmHg diastolic blood pressure.
- Hypotension (< 100 mmHg systolic)
- Severe desaturation: SpO_2_ ≤ 80% when accompanied by symptoms of severe hypoxaemia.
- Sudden pallor.
- Loss of coordination.
- Mental confusion.
- Dizziness or faintness.

### Whole-body FDG uptake using PET/CT methods

Participants will be booked in for their pre-treatment PET/CT scan on Day 11 after baseline at one site.

#### Imaging and multi-organ metabolic changes

Participants will be asked to consent to two whole-body FDG PET-CT scans using the Siemens Biograph Vision (Siemens Healthiness, Germany). Participants will fast for six hours, and blood glucose will be analysed via capillary sampling techniques before F-18 FDG is intravenously injected. The activity of FDG administered for each instance of PET-CT imaging will be based on the participant’s weight (3 MBq/kg) up to a maximum of 400 MBq. The maximum effective dose from one of these administrations would be 7.6 mSv. A parametric (dynamic) protocol will be used for PET-CT imaging and will commence at the time of injection, with the first 6 min positioned over the heart and lungs, and then using the Siemens motion flow protocol up to 1-h post-FDG injection. A standard attenuation correction CT scan at 120 kV with mAs modulation and a PET emission scan using a 3-dimensional acquisition mode will be performed immediately. Fusion PET-CT images will be used for accurate anatomical localisation of the avid FDG uptake of LC lesions. Standardised uptake values (SUVs) and K_i_ will be calculated for major organs and bone marrow. Upon completion of the baseline PET-CT scan, patients will be administered Remdesivir in line with the details highlighted in the treatment regime section and repeat scans will be conducted on day 55. Participants will be able to opt out of the PETCT scans if they wish. Before the scan, a pregnancy test (urine dip) will be performed on those women of childbearing potential (WOCP).

### Trial intervention

All participants receive the antiviral medication Remdesivir via intravenous (IV) infusion over 5 consecutive days. It is licensed for use in patients with COVID-19 who have been admitted to the hospital with acute illness.

Patients receive five IV infusions. The first loading dose is infused over 60 min. Subsequent maintenance doses are infused over a 30-min period. Dosing schedules are guided by the SmPC and will be consistent for each participant.

- Day 1: Loading dose of 200 mg of Remdesivir in a 250 mL sodium chloride 0.9% bag via IV infusion over 60 min.
- Days 2–5: Dose of 100 mg of Remdesivir in a 250 mL sodium chloride 0.9% bag via IV infusion over 30 min.

*Dosage modification:* No dosage modifications are permitted.

*Individual stopping criteria:* If a participant develops clinical evidence of a significant adverse reaction during treatment administration, then the administration can be stopped at the direction of the treating clinician.

#### Treatment phase (days 14 to 22)

Participants will attend their local site for five consecutive days. They will need to attend for approximately 2 h, which includes time for details of any adverse reactions, preparation and delivery of the infusion, plus monitoring post-IV intervention. Refer to Additional file 2: Intervention Flow for further information.

Women of childbearing potential will have a pregnancy test at the start of each session to ensure that they are still eligible.

Research staff will check whether there have been any adverse reactions since their last treatment, if they have had one. This will take approximately 10 min.

Once the IV treatment is completed, the participant will be monitored for a further 30 min. A doctor will be available at all treatment sessions.

Time will be allocated to discuss the next study contact and answer any questions (approximately 30 min).

If for any reason a participant misses a dose of the study drug after treatment has commenced, the participant will be withdrawn from treatment (e.g. participant is unable to attend the clinic, or PI decides to cease IMP). In instances of treatment withdrawal, if willing, the participant will continue with all other scheduled assessments as planned, but no further study drug will be administered.

### Adverse event reporting

Remdesivir will be used outside of its licensed indication. For this trial, it is expected that all adverse events (AEs) that show a potential causal relationship with the IMP (known as adverse reactions [ARs]) are recorded. Other AEs of unexpected severity (in the opinion of and discussion with the PI), or which meet the criteria for a serious adverse event (SAE) should also be recorded. Investigators must seek further information on such adverse events and record details in the patient’s medical notes and on the eCRF. They should be recorded using the Common Terminology Criteria for Adverse Events (CTCAE terms) provided in the National Cancer Institute (NCI) CTCAE v5.0. Severity should be assessed using the NCI CTCAE v5.0 grading. The clinical course of each event should be followed until resolution or stabilisation. 

All AEs and SAEs must be recorded from the time of written informed consent until the last study visit. Safety information will be reviewed for ongoing assessment during TMG and Trial Steering Committee (TSC) meetings as per the trial monitoring plan.

#### Pregnancy reporting

Remdesivir should not be used during the first trimester in pregnancy, and WOCP must use effective contraception during treatment. WOCP will be asked to conduct a pregnancy test at each study visit. There is limited data on pregnancy outcomes (< 300) following exposure to Remdesivir in the second and third trimesters, and clinical experience on the effect of Remdesivir on breastfeeding is limited. Therefore, women who are pregnant or breastfeeding will be excluded from participating in the trial.

### Post intervention

#### Safety blood check (day 27)

Post-intervention, all participants will attend their respective sites for a safety blood check. Pregnancy tests and AE assessments will again be conducted.

#### Post-intervention assessment (day 44 (+ 28 days after treatment phase))

Twenty-four hours before this appointment, patients will be sent all the PROMs to complete as before; EQ-5D-5L, PCFS, SBQ™-LC, MRC Dyspnoea Scale, PDQ-5, GAD-7, FAS, and MFIS as described in baseline.

On arrival at the clinic, pregnancy and AE assessments will be conducted in addition to taking blood for testing biomarkers and inflammatory profiles. Physiological and functional status conducted at baseline will be repeated.

#### Post-intervention CPET (days 51 and 52)

Repeat of days 7 and 8. The participant will return the Visible sensor or, where applicable, the paper-symptom diary to the research team at the end of the visit.

For participants at not have PET/CT scans, the study is now over, and all visits have concluded.

#### Post-intervention PET/CT scan (day 55)

Participants will attend the imaging centre for their post-treatment scan. The procedure, including a pregnancy test and AE assessments, will be a repeat of day 11, after which they will have completed all study visits.

### Withdrawal criteria

Each participant has the right to withdraw from the study at any time. The PIs may withdraw a participant from the study or withdraw aspects of the study (i.e. medication, exercise, or specific scans) if it is determined that the participant’s health is compromised by remaining in the study. All data collected from withdrawn participants will be included in the study report.

A PI may also decide to withdraw a participant from the study or reduce their participation at any time for any reason, including but not limited to:

- Ineligibility (arising during the study or retrospectively having been overlooked at screening).
- An AE/SAE/AR/Suspected Unexpected Serious Adverse Reaction (SUSAR) which results in an inability to continue to complete study procedures.
- Consent is withdrawn.
- A participant reports a confirmed pregnancy as guided by the SmPC.

The reason for withdrawal will be clearly stated (wherever possible) and recorded in the eCRF. If the participant is withdrawn due to an AE related to the study medication or trial assessments, the PI will arrange for a follow-up telephone call until the AE has resolved or stabilised.

If the participant is withdrawn from treatment only, all scheduled assessments will be carried out as planned, but no further study drug will be administered.

If the participant is fully withdrawn from the trial, any required safety checks will still need to be undertaken as planned (e.g. safety bloods at day 27). This will be explained to the participant fully by the research team member documenting the withdrawal.

### Target sample size and justification

A key aspect of this study is to inform progression to the main trial. The sample size is based on the feasibility outcomes of process assessments focused on the red, amber and green (RAG) system (Table 3) that tests against being in the RED zone (unacceptable outcome) based on an expectation of being in the GREEN zone (acceptable outcome) and on the sample size to give high power to reject being in the RED zone if the GREEN zone holds true, using the sample sizes provided in Table 1 in Lewis et al. [27]. Based on treatment fidelity (defined at completion of all 5 sessions), if we assume the upper boundary of the RED zone is 70% and the lower boundary of the GREEN zone is 85%, the sample size required for analysis for 90% power and 5% significance is *n* = 72.

**Table 3:** Progression criteria

Feasibility outcome	Do not proceed to definitive trial (red)	Proceed to definitive trial with protocol amendments (amber)	Proceed to definitive trial (green)
Each site is able to run the study	No sites able	1/2 sites able	2/2 sites able
Recruitment uptake (proportion recruited once marked eligible from screening)	< 25%	25–50%	> 50%
Treatment fidelity (defined at completion of all 5 sessions)	< 70%	70–85%	> 85%
Follow-up at 28 days post IMP	< 65%	65–85%	> 85%
Completion of key outcome measures	< 60%	60–80%	> 80%
Completion of CPETs at 7, 8, 51 and 52 days	< 60%	60–80%	> 80%

### Planned recruitment rate

In the two UK-based centres that will take part in this study, we expect a total of at least 120 patients that will be potentially eligible for the study from established LC clinics and a further 100 from an existing database of patients who have engaged in non-interventional research and have consented to be contacted about relevant research opportunities. Based on 220 potentially eligible participants, if at least 50% agree to participate ((i). GREEN zone), this is an indication that enough patients could be approached to participate in this study. Seventy-two patient-participants will be recruited over 12 months (5–6 per month).

### Statistical analysis plan

A detailed statistical analysis plan (SAP) will be drafted by the trial statisticians and approved by an independent statistician on the TSC before the database lock. The study will be reported following the relevant CONSORT2010 statement extension to pilot and feasibility trials [28].

Baseline characteristics of participants will be summarised descriptively. Loss to follow-up after baseline will be summarised visually via a CONSORT style flow diagram. Baseline characteristics will be subjectively examined to assess potential differences between participants who withdraw, discontinue, and complete the trial. The study process will be descriptively summarised (with 95% confidence intervals). This will include recruitment and retention rates. The proportion of patients who did not meet eligibility criteria will be looked at. Adherence and compliance rates will be examined.

Safety outcome measures with reporting as per the clinical trial of investigational medical product (CTIMP) protocol (Medicines and Healthcare products Regulatory Agency (MHRA) guidelines) will be descriptively summarised. 

In general, the use of hypothesis tests is not appropriate for a feasibility study, as the study has not been powered to address these, and the use of estimates with confidence intervals is preferred to obtain signals of efficacy. Secondary outcome analyses should be considered as hypothesis-generating rather than providing firm conclusions. Key continuous secondary outcomes will be descriptively summarised and analysed using a paired-sample t-test approach to calculate the unadjusted change in scores and the 95% confidence interval using normal linear mixed-effects repeated-measures models. The changes between baseline and each of the follow-up time points will be modelled on time point, with adjustment for baseline and recruitment site.

### End of trial definition

The end of the trial is defined as the last visit of the last participant or the completion of any follow-up and data collection process, and all queries. 

### Data management, confidentiality, and sharing

PenCTU will oversee the management of data for the ERASE-LC trial. Participant screening and outcome data will be entered into REDCap, a web-based data capture system [29], by authorised personnel from participating sites. Access to REDCap is regulated using usernames, encrypted passwords, and two-factor authentication. The system maintains an electronic audit trail and hosts in-built validation mechanisms to ensure data integrity and security, including validation checks at the point of data entry.

To ensure data quality and completeness, the PenCTU data team will regularly monitor the data using validated R scripts [30]. Details of these monitoring and validation activities are outlined in the data management plan (DMP), available upon request. The monitoring process includes post-entry validation checks, completeness of critical data items, surveillance of safety and withdrawal reports, and audit trail monitoring. The data manager will provide ongoing reports to the TMG regarding data quality and completeness throughout the trial.

No direct identifiers will be shared with the trial statisticians, and access to directly identifiable information within the data capture system will be restricted to only users who require access. The trial adheres to relevant data protection legislation, including compliance with the Health Research Authority’s (HRA) GDPR.

Data from the ERASE-LC trial will be retained and accessible for fifteen years post-study completion.

### Access to final trial dataset

After the trial has been reported, the anonymised individual participant data that underlie the results will be available on request from the CI and Sponsor, along with supplementary files as required (e.g. data dictionaries, blank data collection forms, analysis code). Data will be shared with (or access to the data will be provided to) requestors whose proposed use of the data has been approved by the CI and Sponsor, under an appropriate data sharing agreement. It will not be possible to identify participants personally from any information shared.

### Dissemination

The results of this trial will be submitted to peer-reviewed journals for publication as soon as data analysis is completed. Participants will not be identified in any publications. PPIE representatives involved in the trial will support the dissemination of the information into the public domain and to the participants involved in the trial, in an appropriate manner. The findings will be presented at national and international conferences.

Social media: findings will be disseminated and publicised through links with organisations with a large social media presence.

### Governance

The Sponsor for this study, the University of Derby, assumes overall responsibility for the initiation and management of the trial. The Sponsor and Funder are not directly involved in trial design, conduct, data analysis and interpretation, manuscript writing, and dissemination of results.

The CI (Chief Investigator) and co-applicants designed the trial with support from the PenCTU. The PenCTU has been allocated tasks associated with the overall trial and data management, including monitoring. The TMG will meet monthly to review trial progress and ensure appropriate trial management. The TSC will meet every 6–7 months per an agreed set of terms of reference to review the trial’s progress and any serious adverse events and will report to the Sponsor. A Data Monitoring Committee (DMC) was not convened as the study is low-risk and non-randomised.

### Ethics

#### Approvals

Approval has been obtained from the HRA, South Central – Oxford B Research Ethics Committee (REC), Administration of Radioactive Substances Advisory Committee (ARSAC) and MHRA for the trial protocol, and other study documentation (e.g. patient information sheet, informed consent form, general practitioner (GP) letters, study advertisements). The CI will ensure that this study is conducted in conformity with the relevant regulations and with the UK Policy Framework for Health and Social Care Research (2017), which has its basis in the Declaration of Helsinki.

### Public and patient involvement

The protocol and supporting documentation have been reviewed by members of the research teams established Patient and Public Involvement (PPI) group. Their feedback has shaped the design of the study processes. One PPI representative (Ms. Skipper) is a co-applicant and is an active member of the TMG to ensure that the project maintains a patient and translatable approach. Ms. Skipper is an LC patient, a former physiotherapist and has worked alongside the study team to ensure the documentation and study processes are appropriate for patients with LC. They have been extremely involved in ensuring that the study risks are identified and mitigated as much as possible. These include:

- Mitigation of transmission of COVID-19 by testing and symptom reporting, wearing masks and providing adequate ventilation, and conducting cleaning and sterilising procedures before and after each test
- A piloted and tested CPET protocol to reduce the risk of post-exertional symptom exacerbation.
- Actions to limit physical, emotional and cognitive stress:
  ◦ Parking adjacent to the testing and treatment rooms
  ◦ Wheelchairs available
  ◦ Minimum noise levels
  ◦ Reduced distractions
  ◦ Low-level lighting in testing and treatment rooms where possible
  ◦ Rest periods provided between periods of talking
  ◦ To limit fatigue, study questionnaires will be provided 24 h in advance to allow for more time to complete them
  ◦ A space to recline if sitting for long periods
  ◦ Time for participants to get up and for periods of lying or sitting to reduce the incidence of postural tachycardia syndrome (PoTS)

The aim is to develop a study where patients feel safe and willing to participate.

Study progress will be discussed at the PPI Group monthly meetings. Their input will also have a significant impact on the future definitive trial.

## Discussion

LC has a profound impact on the functional status and quality of life for people living with this condition and to date there are no established curative treatments. The ERASE-LC study is a multi-centre feasibility trial of a 5-day infusion of the antiviral drug Remdesivir in patients with a diagnosis of LC. ERASE-LC has been developed with patient safety and comfort at the centre of it with a great deal of support from PPI representatives, ensuring clinically vulnerable participants remain safe and well throughout the trial. This feasibility study will provide data on the acceptability and feasibility of the treatment for patients who are suffering symptoms of LC. The antiviral medication Remdesivir has been selected for this study due to its success in reducing the risk of progression to severe disease in patients during an acute SARS-CoV-2 infection. The outcomes of this and any future study will help many people who have experienced great burden and impact on their lives including an inability to work and reduced quality of life.

The findings of this study will inform the design of a future definitive study to test the effectiveness of the treatment. It will also provide any signals of efficacy. The definitive trial will progress if the progression criteria are met according to the ‘stop-go’ criteria.

## Supplementary Information

Additional file 1. ERASE-LC Study Flow Chart. Flow chart illustrating the patient journey through the study. File format.pdf.

Additional file 2. ERASE-LC Intervention Flow Chart. Flow chart illustrating the details of an infusion visit. File format.pdf.

## Data Availability

Not applicable.

## Abbreviations

6MWT: 6-min walk test
ACSM: American College of Sports Medicine
AE: Adverse event
ALT: Alanine aminotransferase
AR: Adverse reaction
ARSAC: Administration of Radioactive Substances Advisory Committee
BASES: British Association of Sport and Exercise Sciences
BNF: British National Formulary
BPM: Beats per minute
CONSORT: Consolidated standards of reporting trials
COVID-19: Coronavirus disease 2019
CI: Chief Investigator
CRP: C-reactive protein
CPET: Cardiopulmonary exercise test
CTCAE: Common Terminology Criteria for Adverse Events
CTIMP: Clinical Trial of Investigational Medicinal Product
DMC: Data Monitoring Committee
DMP: Data management plan
DSQ-PEM: Modified De Paul Symptom Questionnaire-Post Exertional Malaise
ECG: Electrocardiogram
eCRF: Electronic case report form
eGFR: Estimated glomerular filtration rate
EMA: European Medicines Agency
EOI: Expression of Interest
EQ-5D-5L: EuroQol Group 5 Dimensions 5 Level
ERS: European Respiratory Society
FAS: Fatigue Assessment Scale
FBC: Full blood count
FDG: Fluorodeoxyglucose
FEV: Forced expiratory volume
FVC: Forced vital capacity
FFP: Filtering facepiece
GAD-7: Generalised Anxiety Disorder-7
GDPR: General Data Protection Regulation
GP: General practitioner
HLA-DP: Human leukocyte antigen-DP
HRA: Health Research Authority
IFN-γ: Interferon-gamma
IL: Interleukin
IMP: Investigational Medicinal Product
IV: Intravenous
IOS: IPhone Operating System
ISRCTN: International Standard Randomised Controlled Trials Number
IV: Intravenous
LC: Long COVID
LFT: Liver function test
MEP: Maximum expiratory mouth pressure
MFIS: Modified Fatigue Impact Scale
MHC: Major Histocompatibility Complex
MHRA: Medicines and Healthcare Products Regulatory Agency
MIP: Maximum inspiratory mouth pressure
MRC: Medical Research Council
NCI: National Cancer Institute
NHS: National Health Service
NICE: National Institute for Health and Care Excellence
NLR: Neutrophil-leukocyte ratio
PCFS: Post-COVID Functional Status Scale
PCT: Procalcitonin
PDQ-5: Perceived Deficit Questionnaire
PEF: Peak expiratory flow
PEM: Post-exertional malaise
PenCTU: Peninsula Clinical Trials Unit
PET/CT: Positron emission tomography and computed tomography
PI: Principal investigator
PIC: Participant Identification Centre
PIS: Participant information sheet
PLR: Polymorph-lymphocyte ratio
PoTS: Postural tachycardia syndrome
PPI: Patient and Public Involvement
PROM: Patient-reported outcome measure
QR: Quick Response
RAG: Red/amber/green
RCP: Respiratory Compensation Point
REC: Research Ethics Committee
REDCap: Research Electronic Data Capture
RNA: Ribonucleic acid
RPE: Rate of Perceived Effort
SAE: Serious adverse event
SAP: Statistical analysis plan
SARS-CoV-2: Severe acute respiratory syndrome coronavirus 2
SBQ™-LC: Symptom Burden Questionnaire for Long COVID
SmPC: Summary of product characteristics
SPIRIT: Standard Protocol Items: Recommendations for Interventional Trials
SpO2: Saturation of peripheral oxygen
SUSAR: Suspected unexpected serious adverse reaction
SUV: Standardised uptake value
TAU: Treatment as usual
TMF: Trial master file
TMG: Trial Management Group
TNF-α: Tumour necrosis factor alpha
TSC: Trial Steering Committee
VEGF-D: Vascular endothelial growth factor-D
VO2peak: Peak oxygen uptake
VT: Ventilatory threshold
WHO: World Health Organisation
WOCP: Women of childbearing potential
