# Supplementary material for: An open-label, clinical feasibility study of the efficacy of Remdesivir for Long-COVID
Source: Pilot Feasibility Stud. 2026 May 1;12:87. doi: 10.1186/s40814-026-01823-9 (PMC13281550; doi:10.1186/s40814-026-01823-9)
Supplement: Supplementary file 1 — Additional file 1. ERASE-LC Study Flow Chart. Flow chart illustrating the patient journey through the study. File format.pdf. [file 40814_2026_1823_MOESM1_ESM.docx]

**Additional File 1. ERASE Study flow chart.**

**Recruitment Pathways**

Recruitment via established Long COVID Clinics *(F2F/virtual)*

Recruitment via existing database of contact*s (email/phone)*

Recruitment via self-referral to study website

**Detailed Eligibility Screening and Informed Consent (face-to-face)**

Eligible patients attend detailed screening visit (consent, demographics, medical history, eGFR, LFTs). Baseline visit scheduled.

**Eligibility Confirmation**

Eligibility confirmed upon review of blood test results. Baseline visit confirmed via telephone.

**Screening & Recruitment**

**Study Enrolment & Baseline Assessment (Day 0)**

Participants attend study site and complete baseline assessments (see schedule of assessments).

**Baseline Cardiopulmonary Exercise Test (CPET) (Day 7 and Day 8)**

Participants attend study site and conduct two incremental exercise test 24 hours apart.

**Day 11 – Baseline PET-CT scan with FDG Imaging (Exeter only)**

**Baseline Assessment**

**Post Intervention Assessment (Day 44 / +28 days after IMP)**

Participants attend study site and complete post treatment assessments (see schedule of assessments).

**Post Intervention Cardiopulmonary Exercise Test (Day 51 and Day 52)**

Participants attend study site and conduct two incremental exercise test 24 hours apart.

**End of Study**

**Day 55 – Post Intervention PET-CT scan with FDG Imaging (Exeter only)**

**Post Intervention Assessment**

**Treatment (Days 14-22)**

Participants attend treatment site on 5 consecutive weekdays (see dosage schedule, section 11.6). Flexibility given for IMP to be given on the following Monday to allow five consecutive weekdays.

**Safety Blood Check (Day 27)**

eGFR and LFT as per SMPC.

**Intervention**

**Initial Eligibility Screening (over the phone with verbal consent)**

**Receiving Participant Information Sheet (PIS)**

Patients from Long COVID Clinics and existing database contacted by members of the study team and sent PIS. Patients who self-refer visit website, view PIS, and register interest. Study team call back within 7 days of provision of PIS or receipt of expression of interest form.
