# Supplementary material for: An open-label, clinical feasibility study of the efficacy of Remdesivir for Long-COVID
Source: Pilot Feasibility Stud. 2026 May 1;12:87. doi: 10.1186/s40814-026-01823-9 (PMC13281550; doi:10.1186/s40814-026-01823-9)
Supplement: Supplementary file 2 — Additional file 2. ERASE-LC Intervention Flow Chart. Flow chart illustrating the details of an infusion visit. File format.pdf. [file 40814_2026_1823_MOESM2_ESM.docx]

**Additional File 2: Intervention Flow Chart**

**Notes**

**Data Capture**

**Designated Individual**

**Process**

Call to determine reason if no-show.

Compliance Check

Postdoc Researcher/Study Team

Patient arrives at allocated time.

AE/SAE/AE/SAE/AR/SAR/

SUSARs

Postdoc Researcher/Study Team

Establish changes in details/circumstances/adverse events/since last study contact (Approx. 10 mins)

Research Nurse

Dr available if required

AE/SAE/AE/SAE/AR/SAR/

SUSARs

Preparation for IV infusion

(Approx. 15 mins)

Compliance Check

AE/SAE/AE/SAE/AR/SAR/SUSARs

Research Nurse

Dr available if required

**Dp on hand if required**

Delivery of IV infusion

(30 mins)

Post IV monitoring

(30 mins)

Research Nurse/Postdoc Researcher

Dr available if required

AE/SAE/AE/SAE/AR/SAR/

SUSARs

Record Date/Time and Location on CRF and Study Calendar

Postdoc Researcher/Study Team

Discussion of next study contact

(30 mins)
